# High-resolution XEOL spectroscopy setup at the X-ray absorption spectroscopy beamline P65 of PETRA III

**DOI:** 10.1107/S1600577522007287

**Published:** 2022-08-11

**Authors:** S. Levcenko, R. Biller, T. Pfeiffelmann, K. Ritter, H. H. Falk, T. Wang, S. Siebentritt, E. Welter, C. S. Schnohr

**Affiliations:** a Felix-Bloch-Institut für Festkörperphysik, Universität Leipzig, Linnéstraße 5, 04103 Leipzig, Germany; b Deutsches Elektronen-Synchrotron DESY, Notkestr. 85, 22607 Hamburg, Germany; c Laboratory for Photovoltaics, Department of Physics and Materials Science, University of Luxembourg, Rue du Brill 41, 4422 Belvaux, Luxembourg; ESRF – The European Synchrotron, France

**Keywords:** XEOL, XAS, CuInSe_2_, ZnO, GaN

## Abstract

A newly designed setup to perform steady-state X-ray excited optical luminescence (XEOL) spectroscopy and simultaneous XEOL and X-ray absorption spectroscopy characterization at beamline P65 of PETRA III is described. The XEOL setup is equipped with a He-flow cryostat and state-of-the-art optical detection system, which covers a wide wavelength range of 300–1700 nm with a high spectral resolution of 0.4 nm. To demonstrate the setup functioning, low-temperature XEOL studies on polycrystalline CuInSe_2_ thin film, single-crystalline GaN thin film and single-crystalline ZnO bulk semiconductor samples are performed.

## Abstract 2

X-ray excited optical luminescence (XEOL) spectroscopy is increasingly important to understand the interplay between the optical properties, structure and chemical composition, providing insights into the mechanism of radiative recombination for a wide range of materials. This study demonstrates a newly implemented setup to perform steady-state XEOL and simultaneous XEOL and XAFS characterizations at beamline P65 of PETRA III.

## 1. Introduction

X-ray excited optical luminescence (XEOL) spectroscopy is a useful technique to investigate radiative recombination properties using a synchrotron radiation source (Liu & Sun, 2018 ▸; Sham, 2014 ▸; Rogalev & Goulon, 2002 ▸). The method has been increasingly used to access the optical properties of a wide range of materials and to explore the nature and structural environment of their luminescent centers, including studies of metal clusters (Grandjean *et al.*, 2018 ▸), rare-earth and rare-earth-doped oxides (Soderholm *et al.*, 1998 ▸; Ishii *et al.*, 2013 ▸; Fortes *et al.*, 2014 ▸), and various semiconductor bulk samples (Goulon *et al.*, 1983 ▸; Pettifer & Bourdillon, 1987 ▸), thin films (Sham *et al.*, 1993 ▸; Ishii *et al.*, 2001 ▸) and nanostructures (Larcheri *et al.*, 2006 ▸; Wang *et al.*, 2014 ▸; Murphy *et al.*, 2014 ▸). Initially, XEOL was suggested as an alternative optical detection method for X-ray absorption fine-structure (XAFS) measurements (Bianconi *et al.*, 1978 ▸). Later on, it was discovered that in certain cases XEOL offers element- and site-selective information, especially when combined with transmission (Grandjean *et al.*, 2018 ▸; Goulon *et al.*, 1983 ▸; Pettifer & Bourdillon, 1987 ▸), fluorescence (Ishii *et al.*, 2013 ▸; Wang *et al.*, 2014 ▸; Murphy *et al.*, 2014 ▸) and total-electron-yield (Sham *et al.*, 1993 ▸; Wang *et al.*, 2014 ▸; Murphy *et al.*, 2014 ▸) XAFS measurements. However, in other cases, migration of electronic excitations and various energy-transfer processes preclude any element or site selectivity of the XEOL signal (Rogalev & Goulon, 2002 ▸; Soderholm *et al.*, 1998 ▸). In this context and in view of elucidating the possible origin of the luminescence in the material, it becomes clear that conventional XAFS spectroscopy must support XEOL measurements whenever aiming for site-selective analysis (Boscherini, 2008 ▸). In recent years, XEOL has also been used as an alternative detection channel for mapping and imaging nano­structures with high spatial resolution by applying a synchrotron nanobeam (Steinmann *et al.*, 2020 ▸; Hageraats *et al.*, 2021 ▸; Lin *et al.*, 2020 ▸).

Herein, we discuss the capabilities of a new setup designed at beamline P65 (Welter *et al.*, 2019 ▸) of PETRA III to perform high-spectral-resolution XEOL measurements in wide spectral and temperature ranges. The main advantage of the elaborated setup is that it does not require modification of either the sample alignment or the basic instrument to perform simultaneous XEOL and XAFS measurements (2D-XEOL-XAFS). To demonstrate the setup applicability in the near-infrared (NIR), visible (VIS) and ultraviolet (UV) wavelength ranges, we performed low-temperature hard X-ray XEOL investigations on polycrystalline CuInSe_2_ thin film, single-crystalline GaN thin film and single-crystalline ZnO bulk semiconductor samples, respectively, at fixed X-ray energies. In addition, the simultaneous XEOL and XAFS characterization of a crystalline GaN thin film is also demonstrated.

## 2. Experimental

**Figure 1:**
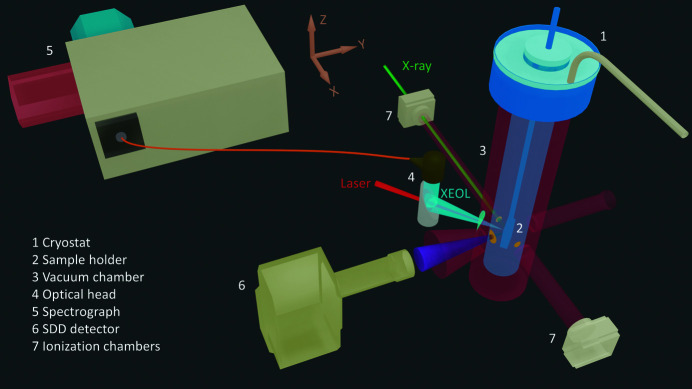
Schematic illustration of the XEOL setup at beamline P65 of PETRA III. The propagation of the X-ray beam is along the *x*-axis direction.

The key elements of the experimental setup for the XEOL measurement are shown schematically in Fig. 1 ▸ and several photographs are given in the supporting information. The design of this setup is constrained by the vacuum requirements for the incoming monochromatic X-ray beam, the transmitted X-ray beam and the X-ray fluorescence to access the full range of X-ray energies provided at beamline P65 of PETRA III at DESY in Hamburg, Germany. The main technical characteristics of this undulator beamline are an X-ray energy range of 4–44 keV, a monochromatic photon flux of up to 10^12^ s^−1^, an energy resolution Δ*E*/*E* of 1.4 × 10^−4^ for Si(111) and of 0.6 × 10^−4^ for Si(311) crystals, and a beam spot size at the sample of 1 mm × 0.5 mm in the horizontal and vertical directions, respectively (Welter *et al.*, 2019 ▸).

To simultaneously measure XAFS spectra and XEOL signals, a modified He-flow cryostat (Janis STVP-FTIR) was manufactured with three X-ray windows made of 25 µm Kapton and located at 180° (incoming X-ray beam, 4 mm diameter), at 90° (X-ray fluorescence, 25° opening angle) and at 0° (transmitted X-ray beam, 4 mm diameter) to the *x*-axis direction, which coincides with the propagation of the X-ray beam (Fig. 1 ▸). The optical window made of quartz is located at 140° (XEOL, 25° opening angle) to the *x*-axis direction. A motorized positioner stage with rotation (360°) around the *z*-axis and translation (60 mm travel distance) along the *z*-axis enables loading several specimens on the sample holder and their adjustment with respect to the incident X-ray beam. In all XEOL measurements reported herein, samples were aligned at the incidence angle of 45°. A temperature controller (Lake Shore 335) maintains a fixed sample temperature in the range 5–300 K with an accuracy of ±0.1 K via a feedback loop with a silicon diode temperature sensor. A six-way cross-type vacuum chamber hosts the cryostat, integrates it into the end-station of the beamline, and provides access to all X-ray and optical viewports. The positioning of the chamber in the *y*-axis direction perpendicular to the X-ray beam is facilitated by a translation stage with 30 mm travel distance in order to align entrance window and sample with the incoming X-ray beam.

The XEOL signal is collected by a 90° off-axis parabolic mirror (Thorlabs, 10 cm focal length) and coupled into an optical fiber with a reflective collimator. Aluminium or silver coating of the mirror and collimator can be selected for optimal signal collection in the UV and NIR-VIS ranges, respectively. These optical elements are the main components of the so-called optical head positioned in front of the optical viewport. In order to simplify the alignment of the optical head, a laser beam is directed at the sample through a center hole in the parabolic mirror. This laser beam can also serve as an excitation source to probe photoluminescence (PL) from the sample. A 633 nm diode laser was used in the measurements reported herein, while a 405 nm diode laser is also available. The alignment of the optical head is optimized with an *xyz* stage with additional rotational freedom around the *z*-axis.

The optical detection system further includes a Czerny-Turner spectrograph (Shamrock 500i) with a 0.5 m focal length, a thermally cooled (Peltier type) linear InGaAs (IGA) array detector (Andor DU490A-1.7, 512 pixels) and a charge coupled device (CCD) camera (Andor DV420A-OE, 1024 × 256 pixels). The triple grating turret of this spectrograph provides the desired band-pass performance and spectral resolution in the region of interest. Two 150 lines mm^−1^ gratings blazed at 1250 and 500 nm and a 300 lines mm^−1^ grating blazed at 500 nm were used in XEOL measurements reported herein. Additionally, the spectrograph has been recently updated with a second turret hosting a 1200 lines mm^−1^ grating blazed at 300 nm, a 600 lines mm^−1^ grating blazed at 500 nm and a 300 lines mm^−1^ grating blazed at 1200 nm, which can be mounted upon user request. A motorized entrance slit (10 µm to 2.5 mm) of the spectrograph allows the intensity and the spectral resolution of the XEOL signal sent through the optical fiber to be adjusted, while a set of suitable long-pass filters mounted on the filter wheel of the spectrograph rejects second-order effects in the acquired spectra. An Hg-Ar light source is used to calibrate the spectrograph and to determine its spectral resolution, defined as the full width at half-maximum (FWHM) of a particular Hg or Ar line. The XEOL raw data are corrected for the spectral response of the detection system using a calibrated halogen lamp as a broadband source.

The X-ray fluorescence detected XAFS spectra were measured in step-wise scan mode (∼355 points) starting 200 eV below the respective edge and ending 700 eV above the edge using a silicon drift detector (SDD). The irradiated sample area was 0.7 mm^2^ and the incoming X-ray photon flux density was 1.4 × 10^12^ s^−1^ mm^−2^ at 9 keV. The time per point was constant over the entire scan range and determined by the counting statistics of the XEOL signal.

## 3. Results and discussion

### 3.1. XEOL measurements

The standard procedure of the XEOL measurement is as follows. The XEOL signal is first measured in the spectral region of interest with the CCD or IGA detector at selected excitation X-ray energies, *e.g.* below and above an absorption edge of interest. In parallel, the *I*_0_ signal is measured at the first ionization chamber which ensures both the monitoring of the incident X-ray beam flux and the normalization of the XEOL intensity probed at each X-ray energy. The XEOL signal can also be measured in the absence of the X-ray beam or even the sample to investigate the presence of various artifacts in the XEOL spectrum due to the scattering light effects in the experimental hutch (Ossig *et al.*, 2021 ▸) or possible emission from the cryostat windows, holders, substrates, *etc*. Once these measurements are completed and typical exposure times are determined, the XEOL and XAFS spectra are detected simultaneously during an X-ray energy scan across the specific X-ray energy region of interest.

### 3.2. NIR-XEOL: a case study on CuInSe_2_

**Figure 2:**
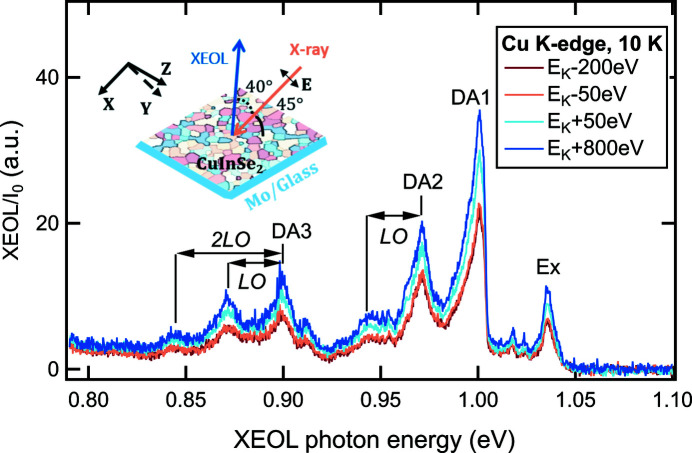
Normalized XEOL spectra measured at several selected X-ray energies across the Cu *K*-edge (*E*_K_ = 8979 eV) at 10 K on a polycrystalline CuInSe_2_ thin film grown on a Mo-coated soda-lime glass substrate. The inset schematically illustrates the polycrystalline character of the sample with color code, the sample orientation relative to the polarization vector **E** of the incident X-ray beam and the direction of the collected XEOL signal. The assignments of the emission bands are based on PL studies (Babbe *et al.*, 2019 ▸; Spindler *et al.*, 2019 ▸), with the definition of the abbreviations given in Section 3.2.

For NIR applications, the 150 lines mm^−1^ grating blazed at 1250 nm and the image area of the IGA detector yield a band-pass, *i.e.* a spectral range or wavelength interval at fixed angle position of the grating, of about 160 nm and a spectral resolution of up to 0.6 nm (0.9 meV at λ = 922.45 nm) for a slit width of 25 µm (Table 1 ▸). This setup configuration has been utilized to study the low-temperature XEOL near the Cu *K*-edge on a polycrystalline CuInSe_2_ thin film sample as shown in Fig. 2 ▸. The CuInSe_2_ layer with a thickness of ∼2 µm was deposited on a glass/Mo substrate using the physical vapor deposition process (Babbe *et al.*, 2019 ▸). To cover a wide spectral range of 0.79–1.10 eV (1127–1569 nm) for the XEOL spectrum, three center wavelengths, *i.e.* three different angle positions of the grating, were necessary. A spectral resolution of ∼2.4 nm (3.6 meV at λ = 922.45 nm) obtained with a slit width of 200 µm and an exposure time of 60 s were used for each center wavelength. This long exposure time is due to the small film thickness (∼2 µm) compared with the much larger absorption length (approximately 12–15 µm) at the Cu *K*-edge in CuInSe_2_. This means that less than 20% of the X-ray flux [an X-ray photon flux density of 1.4 × 10^12^ s^−1^ mm^−2^ (1.4 × 10^14^ s^−1^ cm^−2^)] is absorbed in the sample and, consequently, the excitation density for these XEOL spectra is rather low. The latter is verified with 633 nm laser excitation, showing an intense PL signal with an exposure time of only ∼1 s at moderate illumination (<1 W cm^−2^ or a photon flux density of 3 × 10^18^ s^−1^ cm^−2^). Nevertheless, the characteristic features of Cu-rich CuInSe_2_ PL (Babbe *et al.*, 2019 ▸; Spindler *et al.*, 2019 ▸) are clearly visible in the XEOL spectra presented in Fig. 2 ▸. In particular, the donor–acceptor pair type emissions DA1 at 1.00 eV, DA2 at 0.97 eV and DA3 at 0.90 eV and their longitudinal optical (LO) phonon replicas with a phonon energy of 28 meV as well as the free excitonic emission Ex at 1.04 eV are observed. In addition, there are two weak features near 1.02 eV in the lower-energy region of the Ex transition, which are usually ascribed to the bound exciton transitions (Spindler *et al.*, 2019 ▸). It has been found that the XEOL intensity of the DA and the excitonic emission becomes stronger by about 30% when the excitation X-ray energy varies from 8929 eV (*E*_K_ − 50 eV) to 9029 eV (*E*_K_ + 50 eV) across the Cu *K*-edge. Although XEOL intensity maps with nanoscale spatial resolution measured at 15.25 keV (just above the Rb *K*-edge) have been reported for Cu(In,Ga)Se_2_ solar cells (Ossig *et al.*, 2021 ▸), to our knowledge, we present here for the first time energy-resolved XEOL spectra of chalcopyrite CuInSe_2_ material measured at different excitation X-ray energies.

### 3.3. UV-XEOL: a case study on ZnO

**Figure 3:**
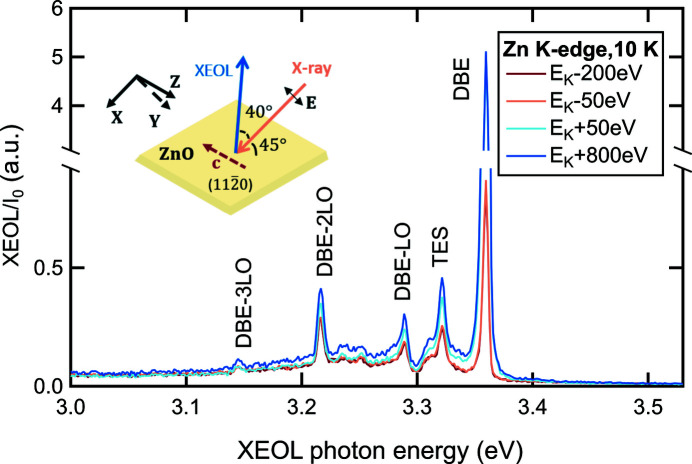
Normalized XEOL spectra measured at several selected X-ray energies across the Zn *K*-edge (*E*_K_ = 9659 eV) at 10 K on a single-crystalline ZnO- 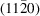 wafer. The inset schematically illustrates the sample orientation relative to the polarization vector **E** of the incident X-ray beam and the direction of the collected XEOL signal. Note that the measurement geometry corresponds to **E** ⊥ **c** (perpendicular) polarization mode. The emission bands are labeled based on PL studies (von Wenckstern *et al.*, 2007*a* ▸,*b* ▸; Wagner *et al.*, 2011 ▸), with the definition of the abbreviations given in Section 3.3.

For measurements in the UV range, the 300 lines mm^−1^ grating blazed at 500 nm and the image area of the CCD detector provide a band-pass of about 170 nm with a spectral resolution of up to 0.4 nm (3.4 meV at λ = 365.01 nm) as indicated in Table 1 ▸. To demonstrate their feasibility, we performed UV-XEOL measurements on a single-crystalline ZnO- 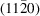 bulk sample at 10 K near the Zn *K*-edge as shown in Fig. 3 ▸. Here we focus only on the near-band-edge (NBE) region of ZnO, even though the measured spectral range extended from 2.66 to 4.23 eV (293 to 466 nm) and the XEOL spectra contained some contribution from the broad defect band centered at ∼2.0 eV. The investigation of this ZnO defect emission has already been reported in the literature (Sham, 2014 ▸; Goulon *et al.*, 1983 ▸; Larcheri *et al.*, 2006 ▸) and is beyond the scope of this work. To enhance the signal-to-noise ratio of some weak luminescence in the NBE region, an exposure time of 20 s is used. Moreover, the ZnO sample is aligned in **E** ⊥ **c** (perpendicular) polarization mode geometry, for which all *A*, *B* and *C* excitonic type transitions originating from the different states of the valence band are allowed (Teke *et al.*, 2004 ▸). The XEOL spectra are composed of the most intense exciton bound to neutral donor (DBE) line at 3.360 eV and its LO phonon replicas with a phonon energy of 72 meV denoted as DBE-LO, DBE-2LO and DBE-3LO lines (von Wenckstern *et al.*, 2007*a* ▸,*b* ▸; Wagner *et al.*, 2011 ▸). The two-electron satellite (TES) transitions of the bound exciton at 3.32 eV are also detected (Teke *et al.*, 2004 ▸; Wagner *et al.*, 2011 ▸). When the X-ray energy increases from 9609 eV (*E*_K_ − 50 eV) to 9709 eV (*E*_K_ + 50 eV), the DBE intensity increases by almost a factor of 6, while the intensity of the TES and DBE-LO lines increases by only a factor of 1.5.

### 3.4. UV-VIS XEOL: a case study on GaN

**Figure 4:**
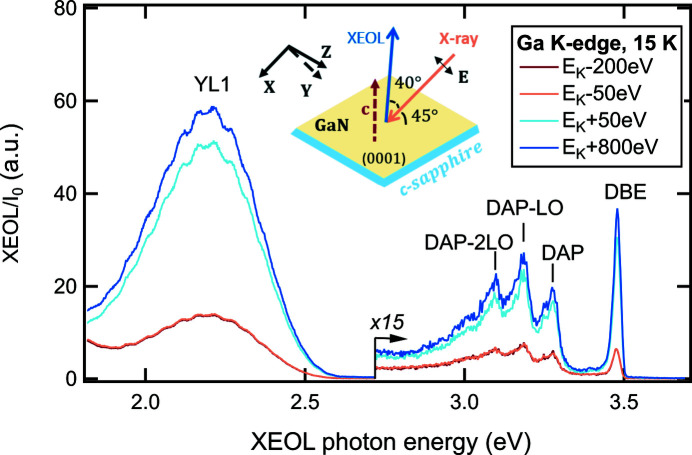
Normalized XEOL spectra measured at several selected X-ray energies across the Ga *K*-edge (*E*_K_ = 10367 eV) at 15 K on a single-crystalline GaN-(0001) thin film grown on crystalline sapphire substrate. The inset schematically illustrates the sample orientation relative to the polarization vector **E** of the incident X-ray beam and the direction of the collected XEOL signal. Note that the measurement geometry corresponds to the mixed polarization case as the incidence angle of the linearly polarized X-ray beam is 45° with respect to the crystal axis *c*. The emission bands are labeled based on PL studies (Reshchikov, 2021 ▸; Reshchikov *et al.*, 2018 ▸), with the definition of the abbreviations given in Section 3.4.

The combination of the 150 lines mm^−1^ grating blazed at 500 nm and the image area of the CCD detector defines the largest band-pass of about 350 nm for the spectrograph (Table 1 ▸). As an illustrative example, this setup configuration enables the monitoring of both the NBE emission and the defect emission of the wide-band-gap semiconductor GaN in the extended photon energy range 1.82–3.69 eV (336–682 nm). The low-temperature XEOL measurements near the Ga *K*-edge (*E*_K_ = 10367 eV) on a 4 µm-thick single-crystalline GaN-(0001) thin film grown on c-Al_2_O_3_ are shown in Fig. 4 ▸. An exposure time of just 1 s per spectrum was obtained with a slit width of 200 µm, however, at the cost of lower spectral resolution of ∼2.4 nm (22 meV at λ = 365.01 nm), which causes an obvious broadening of the UV lines. A spectral resolution of 0.6 nm (6 meV at λ = 365.01 nm) may be achieved by carrying out measurements with 25 µm slit width, which approaches the size of the individual pixel of the CCD camera, however, at the cost of a significantly increased exposure time. The DBE emission at 3.48 eV, the ultraviolet emission presented as a donor–acceptor pair (DAP) band at 3.28 eV and its LO phonon (91.5 meV) replicas, and a yellow defect (YL1) luminescence at 2.20 eV are clearly identified in the XEOL spectra (Reshchikov, 2021 ▸; Reshchikov *et al.*, 2018 ▸). A note of caution is that the strain effects in GaN thin layers grown on sapphire substrates increase the band gap of GaN by 5–20 meV and as a result the spectral positions of luminescence lines are different from those of strain-free GaN samples (Reshchikov, 2021 ▸; Reshchikov *et al.*, 2021 ▸). The small oscillation features with separation of ∼50 meV between the maxima on the band shape of the YL1 emission are most likely caused by the interference effect in the GaN thin layer (Reshchikov, 2021 ▸). Although the DBE emission is relatively weak, it exhibits the strongest increase in intensity by almost a factor of 5 when the X-ray excitation energy is tuned across the Ga *K*-edge, compared with values of 3.5 for the YL1 band and 3 for the DAP bands.

### 3.5. Simultaneous XEOL and XAFS measurements: a case study on GaN

**Figure 5:**
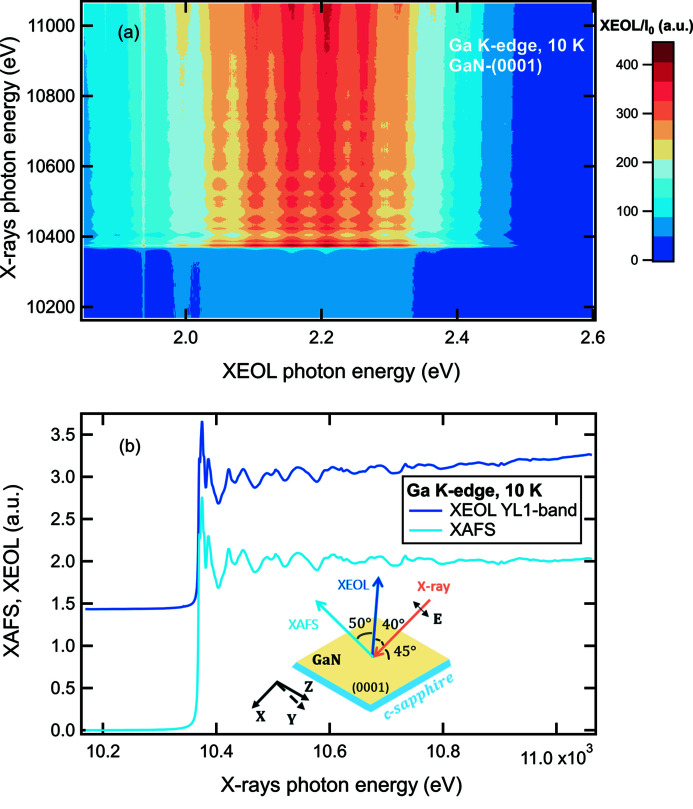
(*a*) 2D-XEOL-XAFS mapping across the Ga *K*-edge (*E*_K_ = 10367 eV) at 10 K on a single-crystalline GaN-(0001) thin film grown on a crystalline sapphire substrate. (*b*) Comparison of the XEOL intensity (offset along the *y*-axis) of the YL1 defect band and the fluorescence XAFS measured simultaneously as a function of the incident X-ray energy. The inset schematically illustrates the sample orientation relative to the polarization vector **E** of the incident X-ray beam and the direction of the collected XEOL and X-ray fluorescence XAFS signals.

The most distinctive advantage of our XEOL cryostat is its ease to simultaneously record XEOL and XAFS spectra in the fluorescence geometry, being applicable to dilute samples or thin films, especially those grown on X-ray opaque substrates. Fig. 5 ▸(*a*) shows an example of the low-temperature 2D-XEOL-XAFS mapping of the YL1 defect band at 10 K on a single-crystalline GaN-(0001) thin film. Details on the X-ray energy range are given in Section 2. The inset of Fig. 5 ▸(*b*) shows the experiment geometry. Note that the intensity of the YL1 defect band is almost independent of temperature in the low-temperature region (Reshchikov, 2021 ▸; Reshchikov *et al.*, 2018 ▸) and the XEOL results obtained at 15 K (discussed in Section 3.4) are also valid here. The normalized XEOL intensity is plotted in a color-coded scheme as a function of both the XEOL photon energy (*x*-axis) and the exciting X-ray energy (*y*-axis) around the Ga *K*-edge. The technical details of this XEOL measurement include a spectral resolution of 0.6 nm (3 meV at λ = 546.08 nm), an exposure time of 3 s and a dwell time of 0.1 s per spectrum. Note that at this exposure time the CCD detector signal (∼6 × 10^5^ counts) almost approached its saturation point (∼6.5 × 10^5^ counts) and, hence, the exposure time can be safely reduced by factor of five or more, still providing good quality data. A horizontal cut through the 2D map in Fig. 5 ▸(*a*) yields XEOL spectra at fixed X-ray energy similar to those shown in Fig. 4 ▸. A strong increase of the intensity for the YL1 band at the X-ray energy of the Ga *K*-edge (*E*_K_ = 10367 eV) is clearly observed in excellent agreement with the spectra plotted in Fig. 4 ▸ for X-ray energies below or above the edge. In contrast, a vertical cut through the 2D map in Fig. 5 ▸(*a*) yields the XEOL intensity at fixed luminescence photon energy as a function of the exciting X-ray energy. As an example, we extracted and normalized the integrated intensity of the YL1 defect band in the 1.9–2.6 eV region (477–653 nm) and plotted it together with the simultaneously measured fluorescence XAFS spectrum in Fig. 5 ▸(*b*). The X-ray energy dependent XEOL spectrum shows not only the edge step at the Ga *K*-edge but also resembles all fine-structure features of the fluorescence XAFS spectrum. Interestingly, our results of a positive edge step for the YL1 band and NBE emission (presented in the previous section, see Fig. 4 ▸) are consistent with the room-temperature polarization-dependent XEOL study of the NBE emission on a 500 µm GaN- 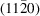 wafer (Lin *et al.*, 2019 ▸), but they are different from earlier measurements at 80 K on a 100 µm free-standing GaN sample, which exhibited a negative edge step and inverted fine-structure features for the XEOL spectra from the blue defect emission at 2.8 eV and the green defect emission at 2.5 eV (Martínez-Criado *et al.*, 2006 ▸). Obviously, further work is needed to resolve these somewhat controversial results on GaN. While this example on GaN was primarily given for the demonstration of the simultaneous XEOL and XAFS measurements with our XEOL setup, it should be pointed out that XEOL experiments on silver clusters in Ag-Linde Type A (LTA) zeolites (Grandjean *et al.*, 2018 ▸), porous Si (Sham *et al.*, 1993 ▸), dopants in TiO_2_:Sm (Ishii *et al.*, 2013 ▸) and Si:Er (Ishii *et al.*, 2001 ▸), ZnO and zinc mesotetra­phenyl­porphyrin (Goulon *et al.*, 1983 ▸), ZnS and ZnSe mixtures (Pettifer & Bourdillon, 1987 ▸) mixtures, and ZnO/CdS core-shells (Wang *et al.*, 2014 ▸) yielded different XEOL-detected XAFS spectra as compared with conventional XAFS measurements performed in fluorescence or transmission modes, thus providing unique information about the nature and local structure of the luminescent centers in these materials.

## 4. Conclusions

The performance of the new high-resolution XEOL setup at beamline P65 of PETRA III has been demonstrated on different semiconductor materials including a single-crystalline GaN thin film and a ZnO bulk sample. We also present the first measurements on the chalcopyrite semiconductor CuInSe_2_ with XEOL spectroscopy. These experiments clearly show that the new setup facilitates the optical characterization of materials over wide spectral and temperature ranges, namely UV-VIS and NIR ranges and 5–300 K, respectively. Furthermore, it easily enables hard X-ray energy-dependent simultaneous XEOL-XAFS measurements. The XEOL setup is available for the user experiments and we expect that future studies will provide valuable information about the radiative recombination properties for a broad range of materials and will enable a more detailed insight into the complex relaxation and energy-transfer processes following the absorption of X-ray photons.

## Supplementary Material

Photographs of the XEOL setup. DOI: 10.1107/S1600577522007287/ok5078sup1.pdf

## Figures and Tables

**Table 1:** Spectrograph band-pass and resolution (Δλ) for different gratings, where λ_blaze_ is the blaze wavelength of the grating. The resolution values are indicated for a slit width of 25 µm

Detector	Grating (lines mm^−1^)	λ_blaze_ (nm)	Band-pass (nm)	Δλ (nm)
IGA	150	1250	160	0.6
CCD	150	500	350	0.6
CCD	300	500	170	0.4
