# Supplementary material for: High-resolution XEOL spectroscopy setup at the X-ray absorption spectroscopy beamline P65 of PETRA III
Source: J Synchrotron Radiat. 2022 Aug 11;29(Pt 5):1209–15. doi: 10.1107/S1600577522007287 (PMC9455204; doi:10.1107/S1600577522007287)
Supplement: Supplementary file 1 [file s-29-01209-sup1.pdf]

JOURNAL OF SYNCHROTRON RADIATION

**Volume 29 (2022)**

**Supporting information for article:**

# High-resolution XEOL spectroscopy setup at the X-ray absorption spectroscopy beamline P65 of PETRA III

**S. Levcenko, R. Biller, T. Pfeiffelmann, K. Ritter, H. H. Falk, T. Wang, S. Siebentritt, E. Welter and C. S. Schnohr**

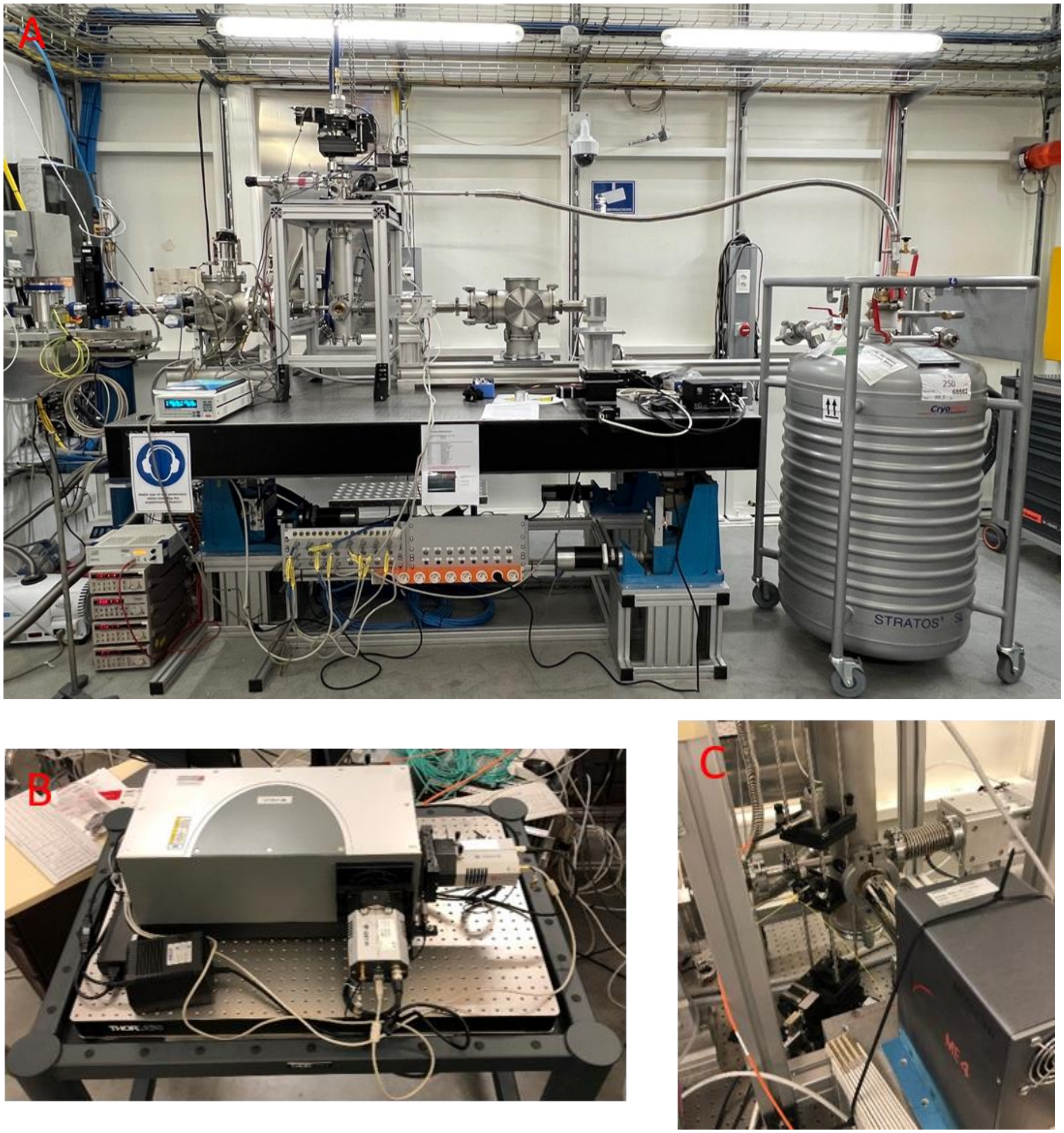


**Figure S1** (A) Photograph showing the He –flow cryostat and vacuum chamber integrated into the end station of the P65 beamline. (B) Photograph showing the optical spectrometer and detectors. (C) Photograph showing the optical head aligned in front of the optical viewport.
